# Structural Biology Illuminates Molecular Determinants of Broad Ebolavirus Neutralization by Human Antibodies for Pan-Ebolavirus Therapeutic Development

**DOI:** 10.3389/fimmu.2021.808047

**Published:** 2022-01-10

**Authors:** Charles D. Murin, Pavlo Gilchuk, James E. Crowe, Andrew B. Ward

**Affiliations:** ^1^ Department of Integrative Structural and Computational Biology, The Scripps Research Institute, La Jolla, CA, United States; ^2^ Vanderbilt Vaccine Center, Vanderbilt University Medical Center, Nashville, TN, United States; ^3^ Department of Pathology, Microbiology and Immunology, Vanderbilt University Medical Center, Nashville, TN, United States; ^4^ Department of Pediatrics, Vanderbilt University Medical Center, Nashville, TN, United States

**Keywords:** antibody, Ebola virus, structural biology, antibody therapeutics, filovirus and viral hemorrhagic fever

## Abstract

Monoclonal antibodies (mAbs) have proven effective for the treatment of ebolavirus infection in humans, with two mAb-based drugs Inmazeb™ and Ebanga™ receiving FDA approval in 2020. While these drugs represent a major advance in the field of filoviral therapeutics, they are composed of antibodies with single-species specificity for Zaire ebolavirus. The Ebolavirus genus includes five additional species, two of which, Bundibugyo ebolavirus and Sudan ebolavirus, have caused severe disease and significant outbreaks in the past. There are several recently identified broadly neutralizing ebolavirus antibodies, including some in the clinical development pipeline, that have demonstrated broad protection in preclinical studies. In this review, we describe how structural biology has illuminated the molecular basis of broad ebolavirus neutralization, including details of common antigenic sites of vulnerability on the glycoprotein surface. We begin with a discussion outlining the history of monoclonal antibody therapeutics for ebolaviruses, with an emphasis on how structural biology has contributed to these efforts. Next, we highlight key structural studies that have advanced our understanding of ebolavirus glycoprotein structures and mechanisms of antibody-mediated neutralization. Finally, we offer examples of how structural biology has contributed to advances in anti-viral medicines and discuss what opportunities the future holds, including rationally designed next-generation therapeutics with increased potency, breadth, and specificity against ebolaviruses.

## Introduction

*Ebolaviruses* are the genus from the family of *Filoviridae* that includes six distinct viral species: Zaire ebolavirus [represented by Ebola virus (EBOV)], Bundibugyo ebolavirus [(Bundibugyo virus (BDBV)], Sudan ebolavirus [Sudan virus (SUDV)], Taï Forest ebolavirus [Taï Forest virus (TAFV)], Bombali ebolavirus [Bombali virus (BOMV)], and Reston ebolavirus [Reston virus (RESTV)]. Of these six species, EBOV, BDBV, and SUDV cause the most severe disease in humans. Together with the related Marburg virus representative of genus *Marburgvirus*, filoviruses have caused at least 30 major, deadly outbreaks since their initial discovery in 1967, with increasing frequency and severity in last decade (1–3). Most filovirus outbreaks are caused by *Ebolaviruses*, which were first described in 1976 (4, 5).

Although the threat of ebolavirus pandemics has loomed for decades, outbreaks tend to be isolated to Sub-Saharan Africa and, in comparison to other human diseases, only affect a tiny fraction of the local populations at the epicenters of these outbreaks. Despite the repeated occurrence of sporadic outbreaks over the past 45 years, no FDA-approved drugs for filovirus infection were approved until 2020. The approval of antibody therapeutics in humans was accelerated due to an unprecedented pandemic that occurred from 2013-2016, when more than 30,000 humans were infected by a novel variant of EBOV (Makona). Two therapeutic interventions currently have FDA approval, both of which consist of monoclonal antibodies (mAbs) and are only effective against EBOV. The single mAb therapeutic named Ebanga™ includes Ansuvimab-zykl (6), and the Inmazeb™ combination of three mAbs includes atoltivimab, maftivimab, and odesivimab-ebgn (7). Ebanga™ and Inmazeb™ were derived from B cells of human survivors or vaccinated, humanized mice, respectively. Both treatments provided significant protection from death and severe disease over the standard of care, according to the results of clinical trials completed during an outbreak that occurred in 2018 (8).

Despite the success of mAb therapeutics for the treatment of autoimmunity and cancer, mAb therapeutics for combatting pathogens have been slow to develop. Other than Ebanga™ and Inmazeb™, there exists only two other antiviral antibody therapeutics with full FDA approval [Synagis, consisting of a single mAb named palivizumab for RSV (9), and Trogazo, consisting of a single mAb named ibalizumab for HIV-1 (10)]. However, the tide is beginning to turn as more antibodies are being isolated and characterized from human survivors, and as animal models and pre-clinical testing are improving and accelerating. For example, the antibody combinations of bamlanivimab plus etesevimab and casirivimab plus imdevimab (11) were both granted emergency use authorization (EUA) in 2020 to treat high-risk patients infected by COVID-19. More recently, a single mAb named sotrovimab was also given EUA authorization for treating COVID-19 (12) and a two-mAb combination of two long-acting mAbs named Evusheld consisting of tixagevimab and cilgavimab was also given EUA recently (13). There are many antibody therapeutics currently in the pre-clinical pipeline for marburgviruses (14–17), dengue (18–20), Zika (21–23), HIV (24–26), influenza (27–29), and coronaviruses (11, 30) (among others).

The severity and size of the ongoing COVID-19 outbreak underscores the importance of having therapeutic prevention at the ready to slow the spread and severity of future epidemics. Despite the existence of two approved therapeutics to treat EBOV, therapeutic gaps remain since these mAbs have only moderate potency for the treatment of severe EVD and would not be effective against other ebolavirus species. The key to developing next-generation antibody therapeutics with improved activity and cross-reactivity is understanding how they act. Insights into mechanism-of-action can be achieved in two principal ways: first, a diverse set of neutralizing and/or protective antibody epitopes recognized by the B cell response of survivors can be mapped to gain insight into sites of vulnerability on the viral surface. Second, high-resolution imaging of antibodies in complex with the viral glycoprotein (GP) can be performed to gain insight into their mechanism-of-action. In this review, we discuss how structural biology has provided insight into the molecular basis of antibody therapeutic efficacy for ebolaviruses, through low-resolution epitope mapping and high-resolution structures of mono-specific or broadly reactive antibodies. These approaches have jointly provided valuable insights into how antibody therapeutics can be improved to develop pan-ebolavirus drugs.

## A Brief History of Antibody Therapeutics for Ebolavirus Disease

Antibody therapeutic intervention has been used to treat ebolavirus infections since the viruses were first described, although the effectiveness of antibody treatment has had mixed success. The earliest example was the transfer of convalescent sera from surviving patients of a mysterious viral disease later identified as MARV, the first filovirus discovered at the time. In that study, all four patients that received sera survived (1–3, 31–33). Later in 1976 when Ebola virus was initially discovered, following the success of the MARV treatments, a plasmapheresis program had limited success, but it was unclear what role plasma played in patient recovery (34, 35). A later trial using convalescent whole blood during the large EBOV outbreak in 1995 involving 8 individuals was highly successful and sparked renewed interest in the use of antibodies as therapeutics for infection (36). Despite the promising results in 1995, these trials were not well-controlled and were difficult to interpret. Later, passive transfer of IgG from a hyperimmunized horse failed to protect macaques, despite a high level of neutralizing titers in the animals (37, 38).

**Figure 1:**
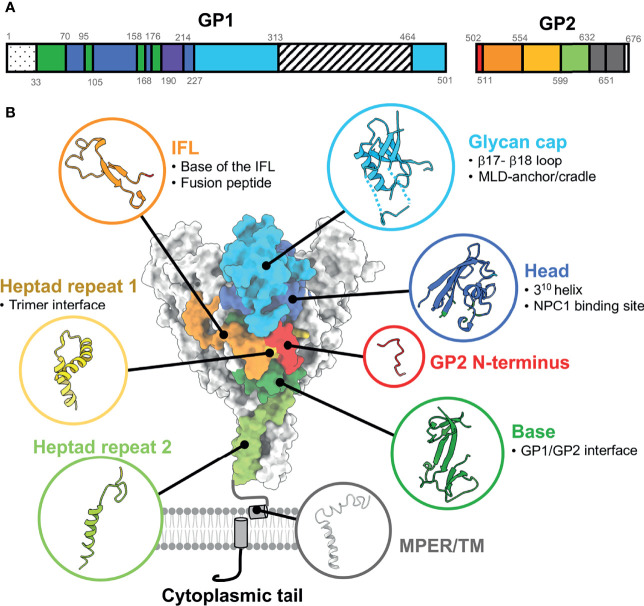
Ebolavirus GP functional and antigenic domain structure. **(A)** Full-length EBOV GP gene schematic indicating domains determined from structural analysis by color. Residues encompassing each domain are indicated by numbers. The 1-33 dotted domain is the non-structural signal peptide. Residues 313-464 are the mucin-like domain (MLD), which is disordered. The purple region from residues 190-214 represents the cathepsin cleavage loop, which hangs over the internal fusion loop (IFL) and is loosely ordered. **(B)** Surface rendering of EBOV GP (PDBID: 5JQ3) with corresponding domains shown in ribbon [based on numbering in part A, from Lee et al. (41)]. Antigenic sub-domains are indicated in bullet points. NPC1, Nieman Pick C1; MPER, membrane-proximal external region; TM, transmembrane domain. The structure of the MPER/TM is from PDBID: 5T42.

One possibility for the mixed response to passive therapy was the lack of control of neutralizing titers and species mismatch of antibodies. In 1999, the generation of recombinant mAbs from survivor bone marrow cells was proposed as an approach to isolate potent antibodies that could be used prophylactically and would also be valuable as analytical reagents (39). One such antibody, named KZ52, was isolated from a phage display library and had moderate neutralizing capability and did not cross-react with the secreted version of the glycoprotein (GP) known as soluble GP (sGP). However, use of the human IgG1 mAb KZ52 as a monotherapy in macaques in 2007 failed to protect animals despite a large dose administered 1 day prior and 4 days after viral challenge (40). *In vivo* escape from KZ52 neutralization was not detected (40). This outcome dampened hopes of antibody therapeutics as viable options for therapeutic intervention in humans. The reason for failure likely was the relatively low neutralizing potency of this antibody. The antibody fragment antigen binding (Fab) of KZ52 did prove vital to solving the first structure of the EBOV GP trimer in 2008 (41). KZ52 binds at the base of GP and likely neutralizes by inhibiting cathepsin-mediated proteolysis of GP (42). Further, the structure of GP provided a means to evaluate vaccines and future therapeutic antibodies by defining structural domains that could be targeted by antibodies (**Figure 1A**).

To help generate potentially higher quality antibodies, many groups turned to vaccinating animals followed by isolating antibodies that exhibited potent *in vitro* properties. Two groups vaccinated mice and created mAbs and performed epitope binning, generating some of the earliest evidence of where sites of vulnerability existed (43, 44). These groups also demonstrated excellent levels of protection in mouse models. Later in 2012, Dye et al. addressed the shortcomings of previous protection experiments by using polyclonal, species-matched antibody transfer in nonhuman primates at delayed and spaced intervals (43). This technique proved to be efficacious by protecting animals from MARV or EBOV up to two days post-exposure. Encouraged by these data, two separate mAb cocktails named ZMab (44–46) and MB-003 (47) were generated using previously developed antibodies from immunized mice. In constructing each three-mAb combination, antibodies were chosen for protection in rodent models and/or *in vitro* neutralizing capacity. For ZMab, this selection included two mouse neutralizing antibodies, designated 2G4 and 4G7, and one non-neutralizing antibody, designated 1H3. For MB-003, this selection included the three non-neutralizing antibodies 6D8, 13F6, and 13C6. Separately, these cocktails provided modest protection in rodent models and monkeys but were later combined into a single preparation called ZMapp™, which included human Fc-chimerized (c) mouse antibodies c13C6, c2G4, and c4G7 (48). These antibodies were individually chosen based on superior protective efficacy in a stringent guinea pig animal model and their combined protective efficacy in non-human primates. Structural evaluation by negative stain electron microscopy (nsEM) indicated that c2G4 and c4G7 recognize overlapping epitopes at the base of GP, very similar to the site recognized by KZ52, but bound to GP at slightly different angles, while c13C6 bound distally to the top of GP in a region known as the glycan cap (49). Importantly, ZMapp™ provided 100% protection in a model of EBOV infection in rhesus macaques, protecting from mortality when administered up to 5 days after viral challenge (48). The development of ZMapp™ coincided with the 2013-2016 EBOV outbreak and was given emergency use approval by the FDA as the outbreak was beginning to wane. Therefore, its effectiveness in humans was not immediately clear due to a limited trial (50).

## Rapid Mapping of Sites of Vulnerability for Protective Antibodies by Negative Stain EM

The availability of a high-resolution GP structure made it possible to evaluate the epitopes of important mAbs in detail (**Figure 1**). In 2011 and 2012, crystallographic studies yielded two more structures of the SUDV GP in complex with 16F6, an antibody very similar to KZ52, but one that was specific for SUDV and bound to GP at a very shallow angle relative to the viral surface (51, 52). Later, in 2015, the first structure of a MARV GP was solved in complex with the neutralizing and protective antibody MR78, also by crystallography (53). Although crystallography has historically been the go-to method for determining antibody-antigen structures, the filoviral GP construct used was particularly refractory to forming crystals without the right antibody Fab bound (54). Further, crystallography did not provide sufficient throughput for analyzing the number of mAbs in need of structural evaluation, which increased dramatically as techniques to isolate mAbs and generate recombinant antibodies advanced. It should be noted that the mucin-like domains (MLDs), which were known to be large, highly glycosylated, and likely unstructured, needed to be removed from crystallographic constructs to help promote crystal formation (55). Later, some structures of antibodies against the MLDs were solved in complex with their linear peptides (55, 56). Generally, however, structures of the MLDs have only been resolved to low resolution (53, 57, 58).

Fortuitously, the technique of single particle EM was rapidly being developed for the evaluation of antiviral antibodies by negative stain methods around the time of the 2013 ebolavirus disease outbreak. In this method, Fab-glycoprotein complexes can be deposited onto carbon-coated grids and stained with a heavy metal salt to reveal white, negatively stained protein particles. The advent of charged couple devices (CCDs) and advanced 3D-reconstruction software made it possible to rapidly take 2D images and process 3D reconstructions in a matter of hours, thus opening the door to evaluating much larger numbers of mAb epitopes. Although these reconstructions are only an average of 15-20 Å resolution, the molecular envelopes reveal enough detail to accurately fit in higher resolution GP structures and models of Fabs, providing a means to easily evaluate their binding sites. nsEM was subsequently used to evaluate the MB-003, ZMab, and ZMapp™ cocktails in 2014, revealing the competing nature of c2G4 and c4G7, c1H3 and c13C6, and the relative location of their epitopes by fitting in the high-resolution crystal structure of EBOV GP (49). Then, in 2015, by fitting the high-resolution crystal structure of MARV GP into a larger cohort of antibodies from a single survivor of MARV infection, structures of several neutralizing antibodies to MARV were also solved by nsEM, indicating they all targeted the highly conserved receptor binding site (RBS) and that it is exposed and accessible for antibody recognition on the MARV GP surface (59).

The 2013-2016 EBOV outbreak required a rapid response to provide emergency use therapeutics necessary to help curb the spread of the pandemic. To help address this need, several groups in parallel pursued the isolation of potent mAbs from human survivors and animal models. A major contributor to the success of this route of therapeutic development was the advent of rapid mAb generation using human hybridoma technology (60). For antibodies that exhibit favorable characteristics *in vitro* (strong GP binding, GP cross-reactivity, potent neutralization), the antibody variable gened in clonal hybridoma cells then can be sequenced, synthesized and expressed to generate recombinant antibodies on larger scales. Prior to and during the outbreak, several groups pursued this route of mAb development and independently generated many different therapeutic candidates (44, 45, 47, 61–63). The Viral Hemorrhagic Fever Immunotherapeutics Consortium (VIC) was also created during this outbreak with the goal of defining correlates of antibody-mediated protection against ebolaviruses and to streamline the process of characterizing therapeutic antibody candidates. The structural biology core of the VIC enabled the side-by-side comparison of many blinded mAbs that had been previously isolated and characterized, further demonstrating the vast immunogenic landscape of the GP (64).

In recent years, the pursuit to identify new therapeutic antibodies has intensified, yielding many new candidates for next-generation therapeutics. These approaches have included the isolation of antibodies from vaccinated animals (65), individuals naturally infected during the 2013-2016 EBOV outbreak (52, 62, 66) or smaller outbreaks of BDBV (61) and long-term survivors of previous outbreaks (67). Additionally, mAbs with favorable *in vitro* and *in vivo* characteristics have been isolated and characterized from humans inoculated with promising vaccine candidates (68, 69), which suggests that these antibodies might provide protection from natural infection. During a more recent EBOV outbreak in 2018, two new mAb combinations were compared head-to-head against ZMapp™, including a three-mAb combination from Regeneron and a monotherapy named mAb114, both of which have gone on to receive FDA approval (see *Introduction*) (8). Importantly, these antibodies have been widely characterized structurally, at least by nsEM, and have shown the extent of vulnerabilities on ebolavirus surface GPs (49, 59, 61, 66, 70–74) (**Figure 1B**). Taken together, these structural data have been valuable for comparing to other viruses, such as HIV, influenza virus, and coronaviruses, where similar efforts have also yielded extensive characterization of sites of vulnerability (75). Thus, efforts to broadly map antibody epitopes for ebolavirus antibodies have yielded more general principles that are conserved across multiple types of enveloped viruses. Importantly, the lessons from these studies have been invaluable for efforts to generate COVID-19 antibody therapeutics and vaccines, rapidly generating effective therapeutics during an active pandemic (11, 30, 76, 77).

## High-Resolution Structures of mABs

Although nsEM is valuable for quickly analyzing antibody binding sties, the inherently low resolution of stain reconstructions prevents a detailed understanding of the molecular basis for how antibodies bind to GP and mediate neutralization, which is where crystallography has been advantageous. However, the filovirus field also rode the wave of the “resolution revolution” in cryogenic EM (cryo-EM) (78), a single particle method that uses protein complexes suspended in vitreous ice within holey grids (79). The lack of a carbon substrate means that electrons passing through the sample are less scattered and therefore can provide more high-resolution details. Advances in techniques for freezing grids, cameras including direct electron detectors, and image analysis software all converged to provide systematic evaluation of many protein complexes at sub-nanometer resolution, often approaching 3 Å resolution or better. Cryo-EM was used in parallel with crystallography, for example, to provide the first structures of the HIV envelope in 2013 (80, 81). Three scientists were later awarded the Nobel Prize in Chemistry for their contributions toward the development of cryo-EM (82), which has revolutionized structural biology and is now the preferred technique for evaluating structures of larger protein complexes.

Cryo-EM has the unique advantage over crystallography of being able to evaluate heterogenous or flexible structures, since regularly ordered protein crystals are not required. Software has been developed to accurately sort complex mixtures of protein structures and states. This development therefore provided a clear opportunity for the filovirus field to apply the rapidity of nsEM with a technique that could provide the same (or better) resolutions of crystallography and virtually any Fab-GP complex. The first cryo-EM structures of EBOV GP were solved in 2016 in complex with the components of the ZMapp™ cocktail, revealing the atomic-level organization of epitope-paratope interfaces and facilitating evaluation of the new variant of EBOV circulating during the outbreak (later named the “Makona” variant) and how it may affect the use of ZMapp™ (70). In parallel, the first and only structure of sGP was also solved by cryo-EM in complex with c13C6 and an sGP-specific mAb, BDBV91 (70). Another group went on to solve the cryo-EM structures of mAb114 and mAb100 in complex with EBOV GP, revealing how mAb114 uniquely accessed the GP head domain (83). Both cryo-EM and crystallography have been used to evaluate many more mAbs, and we will discuss the insights that resulting structures have provided in more detail in the next sections.

## Mechanisms of Ebolavirus Neutralization by Antibodies

The sites of vulnerability on ebolavirus and marburgvirus GPs have been well-established through extensive mapping of protective and neutralizing mAbs. Low-resolution mapping (~20 Å) by nsEM in parallel with fitting high-resolution structures of unliganded GP offers a general idea of where antibodies target to alter the viral lifecycle. Further, the angle at which an antibody Fab binds can be discerned, and polyclonal mixtures of antibodies can easily be evaluated by heterogenous sorting. However, nsEM structures cannot provide reliable details beyond quaternary structure as secondary structural details require sub-nanometer resolution. Faithful docking of Fabs also necessitates *de novo* information regarding structure of the antibodies. Although Fab structures can generally be modeled accurately *in silico*, complementarity determining regions (CDR) loop conformations are notoriously difficult to model accurately, especially for long loops (greater than 21 amino acids). Further, heavy chain (HC) and light chain (LC) assignment is also not possible with most nsEM maps.

Determining a detailed, atomic structure of an antibody-GP complex provides many additional facets of information. First, the regions on GP that are being targeted can be precisely determined, including the structure of paratope CDR loops and the residues that are contacting them. This approach allows for a much more detailed description of how an antibody targets the host virus. Next, high-resolution structures offer a way to interpret the mechanism-of-action of the antibody, whether that be through a direct mechanical interruption of GP function, or potentially though a more subtle mechanism, which can be probed with the added details of atomic structures. High-resolution structures also allow discerning the structural basis for breadth by determining the level of reliance on residues in the epitope and looking at sequence conservation in the context of the structure. Finally, these structures can help pinpoint how naturally occurring mutations in GP may affect antibody therapeutic efficacy. In the realm of vaccines, structure-guided subunit vaccine design has been largely driven by detailed structural information (84–86), which can also be similarly used to guide the design of antibody therapies and pinpoint druggable sites for small molecule design. Below, we provide a detailed description of the major sites of vulnerability of the filoviral GP, with an emphasis on ebolavirus GP, and what structures of GP in complex with antibodies targeting these sites have revealed about mechanism as well as viral biology.

### The Glycan Cap and Mucin-Like Domains

**Figure 2:**
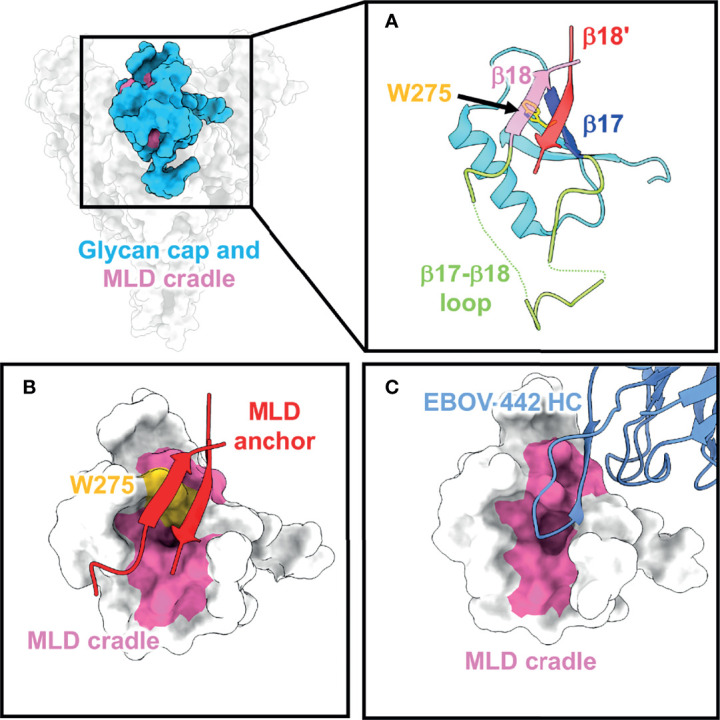
The glycan cap and MLD cradle subdomain. **(A)** The glycan cap (cyan) within the ebolavirus GP consists of globular region that contains several N-linked glycosylation sites, a loosely structured loop (green) between β17 (blue) and β18 (pink) that interacts with the base of the fusion loop in GP2. The mucin-like domains are anchored to the glycan cap *via* β18 and β18′ (red) (PDBID: 5JQ3) and the cradle is subdivided by the critical and conserved residue W275 (yellow). **(B)** The MLD anchor (red), made of β18 and β18′, shields the MLD cradle (pink) and sits over W275. **(C)** The MLD cradle is accessed by broadly neutralizing antibodies through structural mimicry and displacement of the MLD anchor by long CDR loops. Shown is an example from the structure of the broadly neutralizing antibody EBOV-442 (PDBID: 7KFB).

The most N-terminal portion of the GP includes GP1, which is responsible for viral attachment and receptor binding. GP1 is separated from GP2, the region responsible for viral fusion, after furin cleavage occurs in the endoplasmic reticulum approximately between residues 501 and 502 (87–89). On ebolaviruses, an important functional component of GP1 is the glycan cap, a small, structured domain that “caps” the receptor binding site (RBS) by burying residues into the Niemann Pick C1 (NPC1) (90) binding pocket and shielding this region from access by antibodies on GP (**Figure 2A**). The structure of the glycan cap was described in the first crystal structure of GP in complex with KZ52. The portion of the glycan cap C-terminal to the β17-β18 loop was not well-resolved in this structure (41). Later, a higher resolution, unliganded structure of EBOV GP made it possible to build this region more accurately, indicating that a β18-β18′ hairpin structure anchors down the MLDs to the top of the glycan cap in ebolavirus GPs (91) (**Figure 2A**). This finding contrasts with MARV GP, where the “glycan cap” is structured and positioned differently and, consequently, the RBS is fully exposed on MARV GPs (59, 92). It was previously thought that glycan cap antibodies, like MLD antibodies, also would be non-neutralizing since this domain is removed during entry (49). However, a partially neutralizing antibody named 13C6 that was generated in vaccinated mice and later humanized and chimerized with human Fc provided partial protection in animal models and was included in ZMapp™ (47–49). 13C6 can bind bivalently to a single GP spike and possesses antibody dependent cellular cytotoxicity (ADCC) activity due to its Fc-receptor binding activity (47, 70).

The C-terminal portion of the glycan cap is composed of a large, highly flexible region known as the mucin-like domain (41). The “mucin-like” nature of this domain comes from extensive O- and N-linked glycosylation sites that are abundant throughout this region. For ebolaviruses, the MLDs are thought to mainly sit above the glycan cap, while in marburgviruses, they are thought to drape over the sides of the GP (53, 57). Although MLD antibodies have been included in cocktails that provide partial protection in animal models, these antibodies tend to be monospecific due to the sequence heterogeneity of the MLDs (47, 49).

A subset of glycan cap antibodies is known to be neutralizing with a rarer subset possessing potent, pan-ebolavirus neutralizing activity (61, 62, 66, 74). Several broadly neutralizing antibodies all target the same region within the glycan cap. Beneath the β18-β18′ hairpin (known as the MLD anchor) sits a highly conserved patch of residues referred to as the MLD cradle (93) (**Figure 2B**). The cradle forms a hydrophobic pocket centered around the key, completely conserved residue W275, and is accessed by neutralizing antibodies with long CDR loops that mimic the MLD anchor secondary structure to access the cradle (**Figure 2C**). The MLD anchor is loosely tethered and can be displaced. Glycan cap antibodies can also possess variable levels of GP cleavage inhibition *in vitro*, promote trimer destabilization, and enhance the binding of some base-directed antibodies that access the 3 (10) pocket region (described below). The description of pan-ebolavirus, neutralizing, synergistic antibodies has renewed interest in their inclusion in pan-ebolavirus antibody cocktails (73, 93–95).

### Head Domain

**Figure 3:**
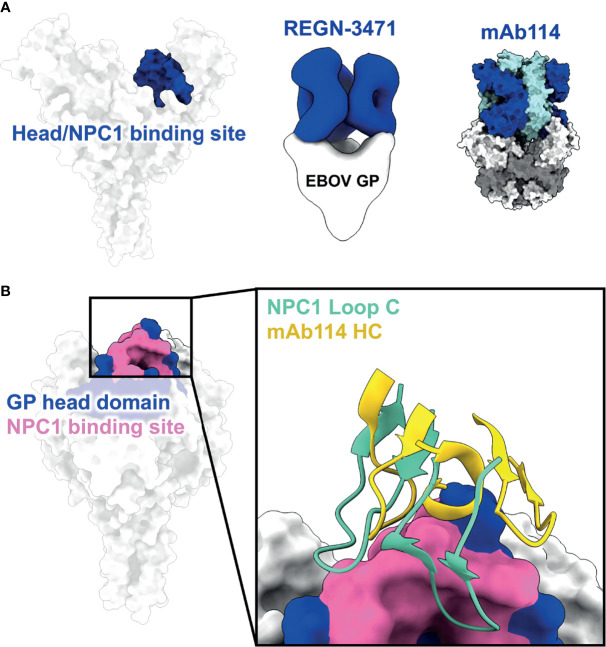
The head domain and NPC1 binding site. **(A)** The head domain on GP sits below the glycan cap. Antibody Fabs of REGN-3471 (left, blue, EMD: 7902) and mAb114 (right, HC in blue and LC in cyan, PDBID: 5FHC) from two FDA-approved EBOV therapeutics access the head domain through very steep angles. **(B)** The NPC1 binding site (pink) necessary for viral entry lies within the head domain (blue). NPC1 binds to this region through its Loop C domain (turquoise), which is partially mimicked and blocked by the CDR loops of mAb114 (yellow).

Beneath the glycan cap, within the core of ebolavirus GP, lies the head domain (*i.e*. the “head” on which the glycan “cap” sits) (41) (**Figure 3A**). Within the head domain is the highly conserved RBS, which includes the NPC1-binding site and is very similar in both ebolaviruses and marburgviruses (53, 96). As described above, the RBS is shielded on ebolaviruses by the glycan cap, which occupies key pockets necessary for access by NPC1 (97–100). However, there have been several antibodies described that can access the small RBS epitope still exposed within the center of the trimer. Two antibodies from the currently FDA-approved therapeutics are known to target this region, including mAb114 (67, 83) and REGN-3471 (65) (**Figure 3A**). What makes these head antibodies unique is that they can still bind with a 3:1 (Fab to GP trimer) stoichiometry despite the tight spacing within the head domain and can simultaneously access both the glycan cap and head domain. Other antibodies also bind in this way or even bind at steeper angles to access the head more extensively, albeit with lower binding stoichiometries (64). This is similar to the antibody FVM04, which also targets the head domain and extends specificity to SUDV (63, 101).

MAb114 stays bound even at low pH (like the pH that would be found in the endosome) and can also bind to EBOV cleaved GP (GP_CL_), meaning that it does not heavily rely on glycan cap contacts (83) (**Figure 3B**). This feature allows mAb114 to block interaction with NPC1, thus this action is likely how mAb114 and similar antibodies mediate their neutralizing activity. To date, no head antibodies have been identified that can also bind to MARV GP, although the inverse is true (there are some head domain MARV GP antibodies that can also bind to EBOV GP_CL_). EBOV head mAbs also are generally specific to a single ebolavirus species. This finding may be a consequence of the mAbs partially contacting some residues of the glycan cap that are less conserved. The head domain itself is clearly accessible, but only the RBS, a smaller and cryptic footprint within the head, is highly conserved across ebolaviruses, while there is also some variability in the rest of the head domain. Therefore, even though the exposed portions of the RBS make them attractive targets for therapeutic antibodies, these antibodies may be inherently limited in breadth. The nature of the epitope also forces Fabs to bind at a nearly perpendicular angle to the viral surface, and they often have excellent Fc-effector activity. However, the connection between the geometry of head-domain antibodies (as well as glycan cap antibodies) and Fc-effector activity is incomplete and should certainly be explored in future studies (102).

### Base/3_10_ Loop and Synergy

**Figure 4:**
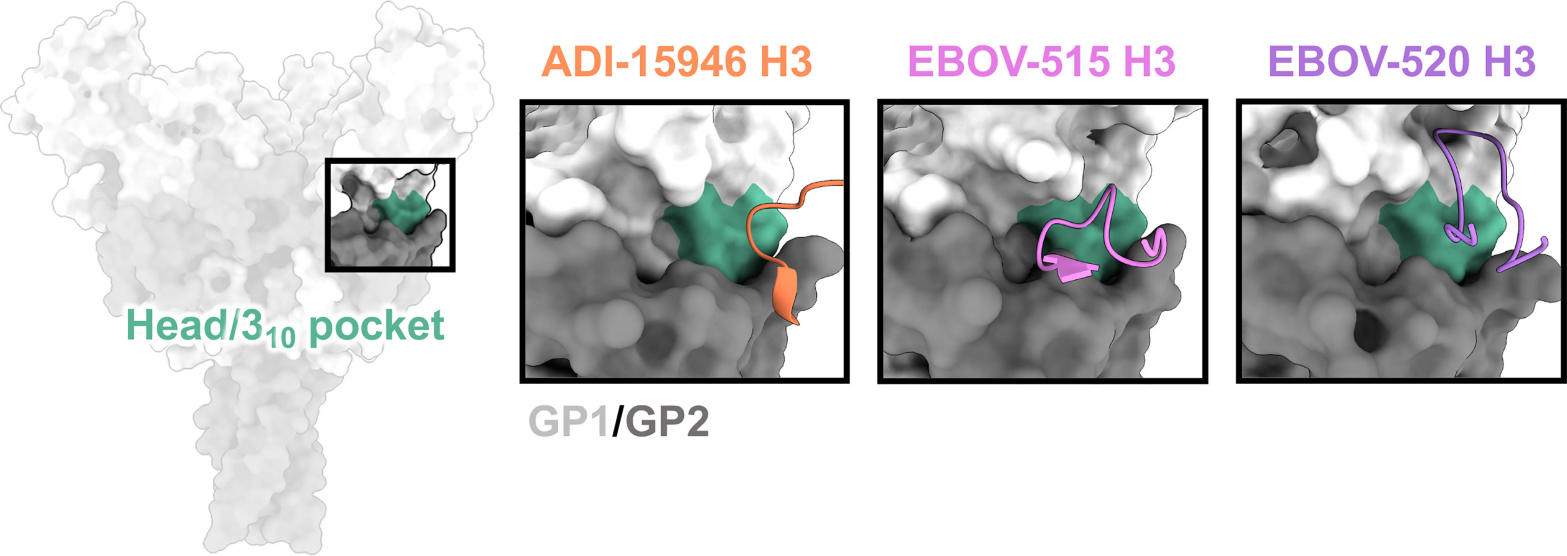
The base and 3_10_ pocket domains. The base domain lies below the glycan cap and above the very bottom of GP, and contains the cryptic 3_10_ pocket (green), which is occupied by the bottom of the β17-β18 loop in unliganded/uncleaved GP. Broadly neutralizing antibodies gain access to the 3_10_ pocket by mimicking and displacing the β17-β18 loop, typically through extended CDRH3 loops. ADI-15946 (orange, PDBID: 6MAM) has a smaller 3_10_ footprint and thus less broad neutralizing activity. EBOV-520 (purple, PDBID: 6PCI) also binds to regions outside of the 3_10_ pocket and thus slightly lower activity toward more divergent ebolavirus species like SUDV. EBOV-515 (pink, PDBID: 7M8L) has the broadest neutralizing activity due to a targeted footprint toward the most conserved portions of the 3_10_ pocket.

On the side of ebolavirus GPs, positioned below the glycan cap and above the base of GP, sits a highly conserved region designated the 3_10_ pocket, composed of residues 71-75 of GP1 that form a 3_10_ alpha helix (71) (**Figure 4**). This domain was first identified in the crystal structure of ADI-15946, a human survivor antibody with high neutralizing potency against EBOV and BDBV, and partial activity against SUDV (71). In the unliganded GP, this pocket is occupied by the β17-β18 loop (91). Antibodies like ADI-15946 can displace the β17-β18 loop to access the 3_10_ pocket. This mechanism was also demonstrated for the neutralizing, pan-ebolavirus human antibodies EBOV-520 and EBOV-515, which similarly access the 3_10_ pocket (95, 103). One feature that is conserved among these antibodies is mimicry of an interaction between W291 in the β17-β18 and N512 at the base of the internal fusion loop (IFL), which forms a loose interaction between GP1 and GP2. These residues are completely conserved in all ebolaviruses. In 3_10_ base-binding antibodies, W291 is mimicked by a tryptophan within the CDRH3, which forms a similar interaction with N512 and displaces the β17-β18 loop. These antibodies bind even more strongly to GP when the β17-β18 loop and the glycan cap are no longer present, such as on GP_CL_.

A unique aspect of some antibodies that bind to the 3_10_ pocket is their synergism with glycan cap antibodies. Both *in vitro* and *in vivo* assays demonstrate that certain glycan cap antibodies enhance binding, neutralization, and protective efficacy of the base antibodies EBOV-520 and EBOV-515 (93, 95, 103). Structural evidence suggests that the displacement of the MLD anchor may loosen contacts between the β17-β18 loop and GP. However, structures of glycan cap antibodies alone bound to GP, for example those of human antibodies BDBV-289 and EBOV-548, show that the β17-β18 loop stays anchored, at least loosely, the base of the IFL even when the MLD-anchor is displaced (93).

Synergy between glycan cap and base antibodies may be assisted by trimer destabilization (93, 95), although it unclear if this occurs on the host cell or viral membranes or how destabilization contributes to synergy or neutralization, if at all. Antibodies toward the surface proteins of other enveloped viruses, including influenza (104–106), HIV (107), and coronaviruses (108), also contribute to trimer destabilization and this mechanism is thought to contribute to neutralization or protection. Antibody synergy has been described for other ebolavirus antibody pairs, such as FVM09 (isolated from monkey) and mC84 (isolated from mouse) (94), which may use a similar mechanism to the EBOV-520/EBOV-548 or EBOV-515/EBOV-442 pairs (93, 103).

### GP1/GP2 Interface and GP2 N-Terminus

**Figure 5:**
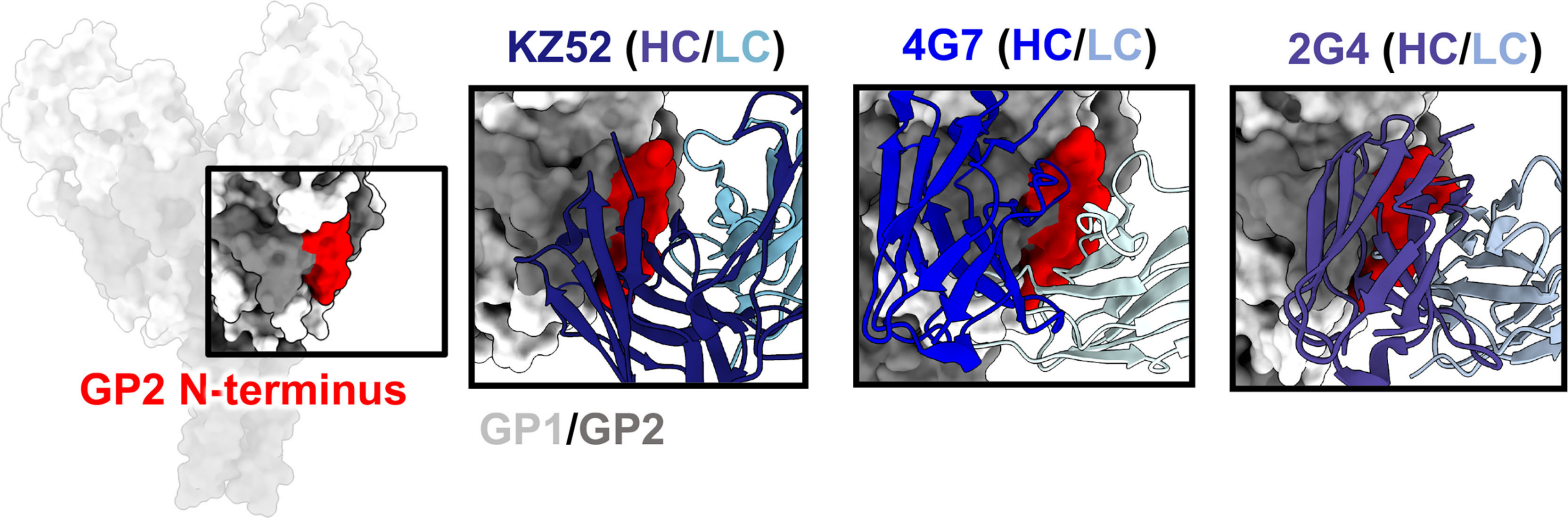
The GP2 N-terminus. The N-terminus of GP2 (red) lies at the interface of GP1 (light gray) and GP2 (dark gray), forming during viral spike processing after furin cleavage, generating GP1 and GP2. This region is a hotspot for neutralizing antibodies, such as KZ52 (left, PDBID: 3CSY), 4G7 (middle, PDBID: 5KEN), and 2G4 (right, PDBID: 5KEL) that access this region in nearly identical ways. A limitation for mAbs binding to this domain is that residues in this region are not well-conserved, and thus mAbs to this site are typically single-virus-species specific.

One of the first GP epitopes to be described in detail was the region targeted by the human phage display library antibody KZ52 (41). KZ52 binds close to the most N-terminal domain of GP1 and interacts directly with the N-terminus of GP2 (**Figure 5**). This same site was later identified as the epitope of two components of ZMapp™, 4G7 and 2G4 (70) (**Figure 5**). Although antibodies such as ADI-15946, EBOV-520, and EBOV-515 have epitopes overlapping with this region, high-resolution structures indicate that the 3_10_ pocket and GP1/GP2 interface represent distinct epitopes within the base of GP (66, 73, 103). It is thought that antibodies that target the GP1/GP2 interface prevent necessary structural rearrangements in heptad repeat 1 (HR1). However, no antibodies that target this region have pan-ebolavirus specificity, all being monospecific. For example, the anti-SUDV antibody 16F6 also binds to the GP1/GP2 interface, although at a very steep angle in relation to the viral membrane (51, 52). However, 16F6 does not cross-react with other ebolavirus species. The reason for limited cross-reactivity may be that the N-terminal portion of GP1 that forms part of the epitope, albeit limited, is necessary for binding. This portion of GP is not well conserved across ebolavirus species and differs quite substantially in MARV GP due to the presence of a wing anchor domain (92). The N-terminus of GP2 also differs in sequence across ebolavirus species. Taken together, the more conserved nature of the 3_10_ pocket over the GP1/GP2 interface makes it a more desirable target for base-binding antibodies included in pan-ebolavirus therapeutic combinations.

### Fusion Loop/HR1

**Figure 6:**
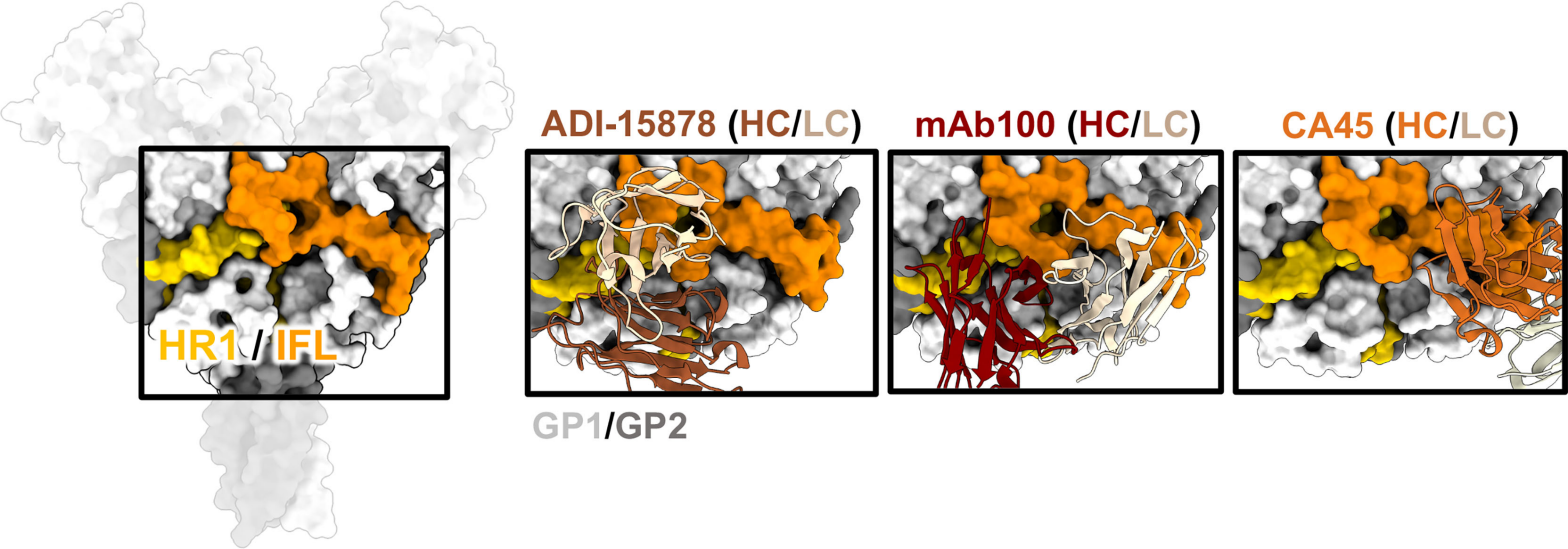
The IFL and HR1. The HR1 domain (yellow) and IFL (orange) form a belt around the middle of GP and antibodies that bind to this region often have overlapping contacts in both these regions. ADI-15878 (left, PDBID: 6DZL) uses a mechanism of induced-fit to mimic GP1 interactions with HR2 and simultaneously access conserved residues in the tip of the IFL. MAb100 (middle, PDBID: 5FHC) binds a very similar epitope to ADI-15878 but has a broader footprint and thus contacts several less-conserved residues making it monospecific. CA45 (right, PDBID: 6EAY) does not contact HR1, focusing more on the base of the IFL and has some broad activity.

A key feature of GP2, the fusogenic domain of GP, is the IFL (**Figure 6**). This loop serves the function of piercing the host cell membrane once receptor binding has facilitated necessary structural changes to release the hydrophobic loop (75). The fusion loop is well exposed on ebolaviruses and highly conserved in sequence. The cathepsin cleavage loop is loosely draped over the base of the IFL and may help to shield access by antibodies and prevent premature springing of GP to a post-fusion state (70, 109, 110). As discussed above, the IFL is also non-covalently anchored to a portion of GP1 at N512 (41). Potentially, this interaction may play a role in the process of fusion by helping to move GP1 domains once the trimer springs.

There have been several mAbs isolated from survivors that bind to the ebolavirus IFL whose high-resolution structures have been elucidated, including CA45 (111, 112), mAb100 (83), and ADI-15878 (71, 72) (**Figure 6**). These antibodies are all neutralizing but CA45 and ADI-15878 possess pan-ebolavirus activity while mAb100 is monospecific for EBOV. The epitopes of ADI-15878 and mAb100 very closely resemble each other since they bind across two GP protomers, contacting HR1 region on one protomer and the IFL on the other protomer (**Figure 6**). However, ADI-15878 is rotated about 90˚ in relation to the mAb100 epitope, placing it within an epitope that includes more conserved residues. Further, ADI-15878 uses an induced fit mechanism to contact its epitope, which likely also assists in focusing contacts on the most conserved residues. In contrast, CA45 binds to the base of the IFL but in a region distinct from 3 (10)/base-binding antibodies.

The mechanism behind IFL/HR1 antibodies is likely mechanical disruption through blocking the release of the IFL. Further, both CA45 and mAb100 inhibit GP cleavage, likely blocking enzymes from accessing the cleavage loop that hangs over the IFL (83, 112). It is unclear if ADI-15878 would also block cleavage since its epitope is a bit more distal from the cleavage loop. In fact, ADI-15946, which binds closer to the base of GP and nearer to the cleavage loop, can simultaneously bind with ADI-15878 (113). More likely, ADI-15878 arrests necessary fusion-activating events. Normally, piercing of the IFL into the host cell membrane would pull the HR1 region, which is attached, into an elongated 3-helix bundle, which is thought to further collapse into a 6-helix bundle with HR2 (114, 115). As pointed out above, ADI-15878 is part of the pan-ebolavirus two-antibody therapeutic cocktail MBP134^AF^ (113, 116), and therefore the IFL/HR1 epitopes are of prime interest for broadly protective antibody therapeutics. Escape mutations in animal models that disable fusion loop antibodies can occur (69), but an antibody that simultaneously contacts both the IFL and HR1 may be less likely to fail in a natural setting since a virus acquiring mutations in both critical regions may be less fit.

## HR2/MPER

**Figure 7:**
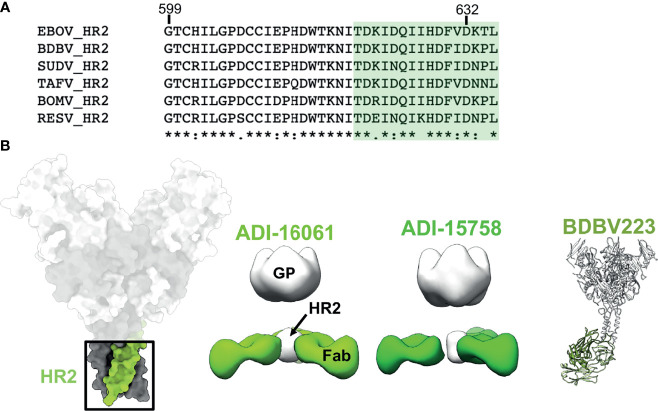
The HR2 domain. **(B)** The HR2 domain (far left in green) lies at the very base of GP, above the membrane proximal external regions that sit directly above the viral envelope and transmembrane domains. ADI-16061 (left, EMD-8698), ADI-15758 (middle, EMD-6588), and BDBV223 (right, composite of PDBID: 6N7J and PDBID: 5JQ3) bind to overlapping epitopes within the HR2 regions, which lies far below the base of GP. Negative stain EM for each of these antibodies indicates that three Fabs can bind simultaneously to a viral spike. **(A)** Sequence alignment of the HR2 region from all known ebolaviruses demonstrating the high level of sequence conservation in this region. The BDBV223 peptide is highlighted in green.

On the C-terminal end of GP2, positioned below the core of GP is the HR2 region followed by the membrane proximal external region (MPER). These features are very common in type I fusion proteins and resemble a type of stalk that anchors the entire viral entry machinery to the viral membrane (75). Consequently, the sequence homology in these domains is highly conserved across ebolaviruses (**Figure 7A**). The HR2 domains form a 3-helix bundle in ebolaviruses and have been described structurally for both liganded and unliganded GP (72, 91, 117). Previous nsEM work completed on a broadly reactive, neutralizing set of antibodies from BDBV survivors demonstrated that, despite the apparent small size of the HR2 epitope, up to 3 antibody Fabs can be accommodated in this domain (**Figure 7B**). Moreover, these antibodies bind much lower down on the spike, below antibodies like KZ52. The HR2/MPER is flexible in relation to the core of the GP, and therefore solving structures of antibodies bound to GP is difficult. A structure of one of these antibodies, BDBV223, in complex with its peptide epitope, demonstrated how antibodies recognize this region (**Figure 7B**) **(** 118). In this case, a single helix is accessed with a limited footprint.

## Broadly Neutralizing Antibodies and Combination Therapies

While the presence of Inmazeb™ and Ebanga™ on the market is an exciting and promising first step toward treating and preventing future EBOV outbreaks, there are several gaps that still need to be filled and improvements that can be made in the field of ebolavirus therapeutics. These drugs both have limitations. First, these mAbs are only effective against EBOV. While EBOV is historically the major cause of the largest outbreaks of filoviruses, there are several other ebolavirus species that are known to be in zoonotic circulation. The possibility of another outbreak of BDBV or SUDV as large at the 2013-2016 EBOV outbreak cannot be overlooked, and the threat of MARV outbreaks also looms. Next, these drugs both require very large doses and therefore are expensive and difficult to produce in the quantities needed to realistically curb a large outbreak. For example, Ebanga was tested at 150 mg/kg in patients and Inmazeb at 50 mg/kg in a clinical trial (8). With an average weight of ~47.0 ± 19.3 kg per patient (across all groups) enrolled in the trial, that dosage amounts to ~2.35 g per antibody per patient (and up to 3.315 g). Conversely, the combination antibody therapy REGEN-COV (casirivimab and imdevimab), which was recently given EUA to treat Covid-19 infection in certain individuals, only requires 600 mg of each antibody. Clearly, improvement of antibody potency *in vivo* is a critical factor of ebolavirus therapeutics that should and will need to be improved in the future.

One way to overcome limited reactivity is to develop alternate antibodies with species cross-reactivity and neutralization. Ideally, a single therapeutic could be developed that can target all three known virulent species of ebolaviruses, retaining equivalent binding, neutralization, and protection for each species. Such pan-ebolavirus antibodies have been actively pursued since the development of the first antibody cocktail ZMapp™. Indeed, cross-reactive antibodies have been shown to be common in the antibody repertoires of EBOV and BDBV survivors (61, 62). A rarer subset of these can neutralize SUDV as well, the most antigenically distinct and historically the most difficult species to target. Structural biology has illuminated the basis for these mechanisms, helping to usher in the creation of next-generation, pan-ebolavirus antibody cocktails (66, 71–73, 93, 95, 103). The base epitope, containing the 3 (10) pocket, the IFL/HR1 region, and the MLD cradle are all hotspots for broadly and potently neutralizing antibodies (whose mechanisms are described in detail above). The way such antibodies specifically target each of these domains allows engagement with crucially important domains and residues that are highly conserved throughout the ebolaviruses. Further, reliance on less conserved residues is low, meaning that some mutations could potentially be tolerated.

The use of antibody cocktails could be considered less desirable than a single antibody for manufacturing reasons. Cocktails require 2-3× more antibodies and are more difficult to develop and acquire approval. However, one potential advantage is the avoidance of escape mutations. With a single antibody, only a single escape mutation may be required to eliminate binding, as has been recently seen with bamlanivimab treatment for SARS-CoV-2 infections (119, 120). For a combination, however, even if a virus escaped one antibody, the development of escape mutations for two or three different broadly neutralizing epitopes is less likely. One argument is that any single member of existing antibody combinations cannot provide potent protection alone (98, 109). However, there is not currently evidence to suggest that a loss in potency due to escape mutation from a single component of a cocktail would result in therapeutic failure. In addition, individual mAbs of recently identified pan-ebolavirus two-antibody combinations MBP134^AF^ or EBOV-442/EBOV-515 conferred high level of therapeutic protection at least against EBOV.

There are currently two leading candidates for pan-ebolavirus combinations based on pre-clinical studies in non-human primates, including MBP134^AF^ and EBOV-442/EBOV-515, which were derived from human B cells following natural human infection (66, 103, 113, 116). Both combinations are composed of only two antibodies, broadly neutralize EBOV, BDBV, and SUDV, combine neutralizing and Fc-mediated antiviral mechanisms, and provide complete protection in animal models for all three viruses, even when given several days after infection onset of severe symptoms. Each combination also has its own distinct features. For example, between the comparable antibodies ADI-23774^AF^ and EBOV-515, which both target the 3 (10) pocket, ADI-23774^AF^ was generated *in vitro* from a library and affinity matured, while EBOV-515 was isolated directly from natural infection. Maturation of ADI-23774^AF^ was necessary to increase binding and neutralization potency toward SUDV, while EBOV-515 already neutralizes SUDV potently and binds to GP even at low pH (103). Also, EBOV-442/EBOV-515 antibodies are differentially tuned for Fc function, in which EBOV-515 principally acts *via* direct virus neutralization and EBOV-442 possesses Fc-mediated effector activity in addition to neutralizing activity. In the MBP134^AF^ cocktail both mAbs are afucosylated variants for enhanced Fc activity. Regarding dosing, MBP134^AF^ has been shown to be effective with a single, low dose (25-mg/kg) in non-human primate model of ebolavirus infection while EBOV-442/EBOV-515 has only been tested in two doses at 30-mg/kg on days 3 and 6.

Another reported feature of EBOV-442/EBOV-515 is that these antibodies exhibit synergy that is mediated by structural GP remodeling after antibody binding (103). Synergistic *in vivo* activity has been also suggested for MBP134^AF^ although the mechanism for this activity is unknown. Indeed, synergy in polyclonal antibodies may be a very common mechanism for surviving natural infection, as we and others have demonstrated, including for viruses other than ebolaviruses (93–95, 103, 108, 121–124). Synergy could also lower the required amount of each antibody needed, although dosing and pharmacokinetic studies first need to be carefully developed in humans. MBP134^AF^ and EBOV-442/EBOV-515 both demonstrated excellent protection in stringent rodent models and non-human primates (103, 116). However, a head-to-head comparison in NHP models has not yet been performed and a clinical trial, like the PALM study (8), would also need to be performed to determine if there are any true advantages of one over the other in protecting humans from severe disease and mortality.

An additional and promising way of using antibody combination therapies is to generate bispecific antibodies that can incorporate multiple specificities within a single antibody treatment. This strategy was used by a group that sought to target antibodies to cleaved GP, which is highly conserved and been considered as a potential vaccine target. Unfortunately, the most conserved regions are not accessible on the surface of ebolavirus GP but are exposed once the virus is endocytosed and cleaved. By combining the antibody FVM09, which targets a highly conserved region in the glycan cap to deliver the antibodies to the endosome, with antibodies against either NPC1 or the RBS, bispecific antibodies could broadly neutralize all ebolaviruses and even provided significant protection in animal models (125). To overcome potential escape from FVM09, this same group later developed next-generation bispecific antibodies that target the endosomal pathway by binding to internalizing, cell-surface receptors (126). Continued development of these therapeutics may be warranted and could reveal exciting alternatives to single antibody or combination therapies.

## Concluding Remarks

Therapeutic antibodies for the treatment of cancer and autoimmunity have long been an effective strategy for treating these diseases. Now, antibody therapeutics for the treatment of viral infections are entering a renaissance phase in the medical community as well. For filoviruses, we may see new licensed antibodies soon for marburgviruses (14) and for pan-ebolavirus treatment (103, 116). Structural biology has been a valuable tool for the development of these and other antibody therapeutics, both by defining sites of vulnerability and by providing atomic level details of antibody mechanisms-of-action. While mAbs have been paramount for structural biology efforts, and necessary to generate therapeutics, they are highly selected and do not reflect the full humoral response during natural infection. EM-based polyclonal epitope mapping (EMPEM) is a valuable new tool for studying the full antibody repertoire in survivor serum at various stages of infection, or as a tool for evaluating vaccines (127–129). Mapping polyclonal serum antibody specificities by EMPEM paints a more complete picture of the human immune response to filoviruses, and high-resolution cryo-EM studies could complement these efforts to reveal the more specific and subtle epitopes that are being accessed during natural infection or vaccination but have been missed by mAb studies (130, 131). Such studies will be helpful in tuning vaccine design and for selecting antibodies that result from protective humoral responses.

There is still much to be learned regarding the contribution of Fc effector functions to the protective efficacy of antibodies for filoviral infection. There are several lines of evidence that suggest that Fc effector function can enhance the protective potency of some antibodies in a combination. However, correlates of protection are difficult to assess with isolated mAbs, and a detailed role for Fc-related mechanisms in protection against disease during natural infection or vaccination in humans is not understood. Anecdotally, antibodies that target the top of the GP spike such as the EBOV antibody 13C6 tend to exhibit antibody dependent cellular cytotoxicity (ADCC) *in vitro*. Future studies to address the molecular mechanism of ADCC, for filoviruses and other enveloped viruses, may reveal new clues that could help develop antibody therapeutics with more potent and targeted Fc effector functions. Non-diffraction limited microscopy technologies, such as super-resolution light microscopy [for example, MINFLUX (132)], as well as cryo electron tomography (133, 134), are powerful tools for studying biologically relevant systems. As these technologies develop and improve, we can harness their power to gain a more in-depth insight into how immune complexes form on the surfaces of infected cells and viruses and subsequently interact with host effector cells and receptors, thus guiding antibody therapeutic engineering efforts.

## Author Contributions

CM wrote the manuscript and generated the figures. PG, JC, and AW helped write and edit the manuscript. All authors contributed to the article and approved the submitted version.

## Funding

This work was supported by the NIH (U19 AI142785 and U19 AI109711).

## Conflict of Interest

JC has served as a consultant for Luna Biologics and Merck Sharp & Dohme Corp., is a member of the Scientific Advisory Board of Meissa Vaccines and is Founder of IDBiologics. The Crowe laboratory at Vanderbilt University Medical Center has received sponsored research agreements from Takeda Vaccines, IDBiologics and AstraZeneca.

The remaining authors declare that the research was conducted in the absence of any commercial or financial relationships that could be construed as a potential conflict of interest.

## Publisher’s Note

All claims expressed in this article are solely those of the authors and do not necessarily represent those of their affiliated organizations, or those of the publisher, the editors and the reviewers. Any product that may be evaluated in this article, or claim that may be made by its manufacturer, is not guaranteed or endorsed by the publisher.
